# Direct Synthesis of Co-doped Graphene on Dielectric Substrates Using Solid Carbon Sources

**DOI:** 10.1007/s40820-015-0052-6

**Published:** 2015-07-16

**Authors:** Qi Wang, Pingping Zhang, Qiqi Zhuo, Xiaoxin Lv, Jiwei Wang, Xuhui Sun

**Affiliations:** Jiangsu Key Laboratory for Carbon-Based Functional Materials and Devices, Institute of Functional Nano & Soft Materials, Soochow University, Suzhou, 215123 Jiangsu People’s Republic of China

**Keywords:** Graphene, Solid carbon sources, Transfer-free, Doping and co-doping

## Abstract

Direct synthesis of high-quality doped graphene on dielectric substrates without transfer is highly desired for simplified device processing in electronic applications. However, graphene synthesis directly on substrates suitable for device applications, though highly demanded, remains unattainable and challenging. Here, a simple and transfer-free synthesis of high-quality doped graphene on the dielectric substrate has been developed using a thin Cu layer as the top catalyst and polycyclic aromatic hydrocarbons as both carbon precursors and doping sources. N-doped and N, F-co-doped graphene have been achieved using TPB and F_16_CuPc as solid carbon sources, respectively. The growth conditions were systematically optimized and the as-grown doped graphene were well characterized. The growth strategy provides a controllable transfer-free route for high-quality doped graphene synthesis, which will facilitate the practical applications of graphene.

**Electronic supplementary material:**

The online version of this article (doi:10.1007/s40820-015-0052-6) contains supplementary material, which is available to authorized users.

## Introduction

Graphene, a one-atom-thick layer of carbon with *sp* ^2^ hybrid orbital bonding and two-dimensional structure material, has attracted intense research interests due to its extraordinary physical and chemical characteristics, such as good mechanical strength [1], high carrier mobility [2], excellent electrical conductivity [3], superior thermal conductivity [4], and high transmittance [5]. However, the nature of pristine graphene with zero band gap brings some difficulties for its application in the electronic device field [6]. Doping of graphene with other heteroatoms (e.g., nitrogen, boron, phosphorus, halogen, etc.) is the most practicable, convenient, and efficient approach to modulate the band structure and properties of graphene [7] and further extend more useful applications in electronics and electrochemical cells [8–13].

Among all the approaches to synthesize doped graphene, chemical vapor deposition (CVD) is the most popular method to obtain high-quality doped graphene in large scale by introducing copper or nickel foil as the catalyst [3, 14, 15] and independent doping source (e.g., NH_3_ as N doping source) [16, 17]. Recently, carbon sources containing dopant element have been used to directly grow doped graphene by CVD method, avoiding the post-doping treatment or using dopant gases in the growth process. For example, Tour et al. demonstrated a new approach that large area, high-quality N-doped graphene with controllable thickness can be grown from different solid carbon sources such as polymer films or small molecules, deposited on a metal catalyst substrate at 800 °C [18]. Liu et al. developed a self-assembly approach that allows the synthesis of single-layer and highly nitrogen-doped graphene domain arrays by self-organization of pyridine molecules on the Cu surface [9]. However, the graphene film obtained by these methods generally requires physical transfer onto the desired substrates for subsequent device processing [19, 20], which could introduce the defects and contaminations into the graphene film.

Recently, we have developed a new transfer-free approach capable of synthesizing graphene directly on dielectric substrates using polycyclic aromatic hydrocarbons (PAHs) as carbon sources [21]. Significantly, N doping and patterning of graphene can be readily and concurrently achieved by this growth method. In this paper, we systematically investigate the factors that affect the growth quality of the doped graphene and optimized the growth conditions for high-quality doped graphene. Furthermore, we demonstrate that N, F-co-doped graphene can be synthesized using only 1, 2, 3, 4, 8, 9, 10, 11, 15, 16, 17, 18, 22, 23, 24, 25-Hexadecafluorophthalocyanine Copper(II) (F_16_CuPc) as solid carbon source and both N and F doping sources.

## Experimental Section

**Fig. 1:**
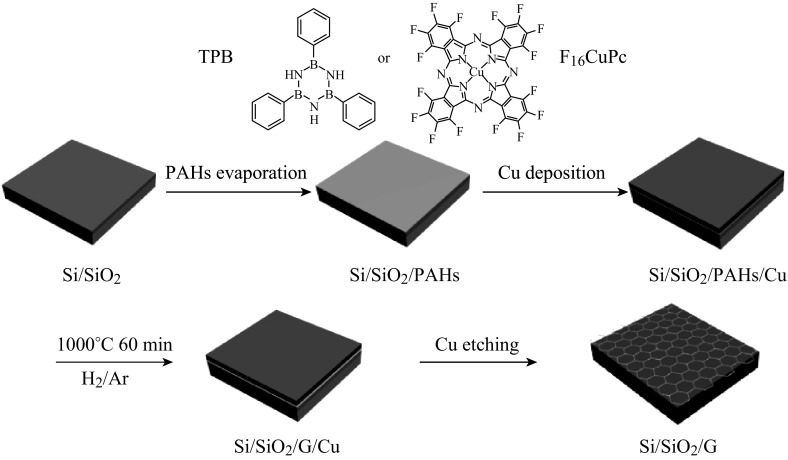
Schematic representation of the doped graphene synthesis process

The schematic of growth process of doped graphene directly on SiO_2_-layered Si (SiO_2_/Si) without transfer is shown in Fig. 1. First, the SiO_2_/Si (SiO_2_: 300 nm thick) substrate was ultrasonically cleaned by acetone, ethanol, and deionized water for 15 min, respectively. Then PAHs with planar structure (TPB or F_16_CuPc) were evaporated on the substrate as solid carbon sources by thermal evaporation system (Organic Evaporation Coating Machine ZZB-U500SA), followed by the Cu film layer deposition on the surface of PAHs as catalyst by electron-beam evaporation system (Kurt J. Lesker, PVD750). After annealing in a tube furnace under Ar gas flow at ~1.8 × 10^2^ Pa, doped graphene was synthesized between the Cu layer and the substrate. At last, Cu layer was etched away by Marble’s reagent (CuSO_4_:HCl:H_2_O = 10 g:50 mL:50 mL), then doped graphene was obtained directly on SiO_2_ substrate without any transfer process.

The morphology of doped graphene was characterized by scanning electron microscopy (SEM) (FEI Quanta 200F). Raman spectra were recorded at room temperature using a Jobin–Yvon HR800 Raman microscope with laser excitation at 514 nm. Optical images were obtained using Fluorescence optical microscope (DM4000M). The HR-TEM images were taken by transmission electron microscope (TEM) (Tecnai G2 F20). The surface state and electron structure of the samples were studied by X-ray photoelectron spectroscopy (XPS) measurement (Kratos AXIS UltraDLD ultrahigh vacuum (UHV) surface analysis system), using Al *K*α X-rays (1486 eV) as the excitation source. The optical transmittance spectrum and sheet resistance of doped graphene are measured by ultraviolet–visible–near-infrared spectrophotometer (LAMBDA 750) and four-point probe system (ST-2258A), respectively.

## Result and Discussion

Carbon source is an important factor in graphene synthesis. We found that planar configuration of PAHs might provide a hexagonal honeycomb skeleton structure for the graphene growth and the growth mechanism from PAHs maybe involves surface-mediated nucleation process of dehydrogenated PAHs catalyzed by Cu rather than segregation or precipitation process of small carbon species that decomposed from the precursors. Therefore, planar PAHs that contain heteroatoms (e.g., nitrogen, boron, fluorine) were chosen as solid carbon sources for doped graphene growth in our work. In addition to the specific structure of solid carbon sources, there are some other key factors to control the quality of doped graphene, such as the thickness of solid carbon sources, the thickness of Cu film layer, annealing time, annealing temperature, etc. Hence, in order to achieve high-quality doped graphene, optimal conditions for doped graphene growth had been investigated by rationalizing the above factors. 2, 4, 6-triphenylborazine (TPB) with planar configuration was selected as the solid carbon source to evaluate the growth conditions of the doped graphene.

The thickness effect of TPB on the quality of graphene was investigated firstly. The Raman spectra shown in Fig. S1a reveal that the optimum thickness of TPB layer is 5 nm. When the thickness of TPB is less than 5 nm, the carbon source cannot form continuous film on the substrate, which could result in the formation of discontinuous graphene. While the thickness of TPB is greater than 5 nm, the excessive amount of carbon source leads to multilayer graphene or amorphous carbon formation due to the extremely low solubility of C in Cu. Different annealing temperatures were also investigated for the growth of TPB-derived doped graphene. In general, the growth temperature, in conventional CVD method, required to synthesize good-quality graphene is 1000–1050 °C. Figure S1b shows the Raman spectra of graphene synthesized at different growth temperatures, suggesting that graphene can be obtained above 950 °C. Annealing temperature below 650 °C results in the deposition of amorphous carbon, as characterized by the broad D and G bands and a very weak 2D band shown in Fig. S1b. When the annealing temperature was increased to 1050 °C, the obtained graphene layer also has a larger D band than that grown at 1000 °C in the Raman spectra. It has probably arisen from the partial evaporation of thin Cu film at 1050 °C.

Subsequently, different annealing times were studied. As shown in Fig. S1c, higher quality doped graphene with lower *I*_D_/*I*_G_ and higher *I*_2D_/*I*_G_ ratio can be achieved when the annealing time is 60 min. The effect of Cu film thickness on doped graphene growth was investigated as well. When Cu film thickness is above 100 nm, the graphene film can be obtained. However, when the thickness of the Cu film was decreased below 100 nm, most of the Cu was evaporated during the annealing process at 1000 °C and resulted in discontinuous doped graphene. In addition, the graphene formed on the top surface of Cu was observed when a thin Cu layer was used. When the thickness of the Cu film was increased to 1000 nm, relatively high-quality doped graphene was obtained indicated by the G/2D ratio (~0.3), D/G ratio (~1.3), and FWHM of the 2D (~42 cm^−1^) band in Fig. S1d. Thus the optimal growth conditions for the doped graphene growth from TPB were set at 5 nm TPB as carbon source, 1000 nm Cu film on the top surface, and annealing temperature of 1000 °C for 60 min.

**Fig. 2:**
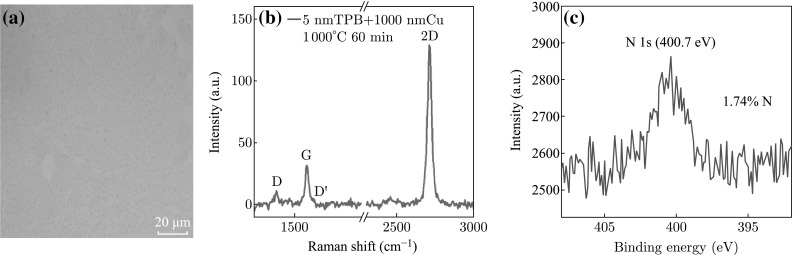
**a** Optical image, **b** Raman spectrum, and **c** the high-resolution XPS scan of N 1s of doped graphene grown from 5 nm TPB, 1000 nm Cu, annealing at 1000 °C for 60 min

Figure 2a shows the optical image of the doped graphene grown on SiO_2_/Si substrate at the optimal condition using TPB as the carbon source. The continuous film with almost no contrast indicates that the graphene is distributed uniformly on the dielectric substrate. The corresponding Raman spectrum in Fig. 2b shows a weak D band, revealing that the graphene film is almost defect free and the weak D band may have arisen from the doping effect. The G/2D ratio is ~0.25 and the 2D peak is sharp and symmetric, indicating that the obtained graphene is monolayer [22, 23]. The monolayer graphene is also confirmed by AFM measurement as shown in Fig. S2. A small D’ band beside G band confirms that doped graphene has been obtained. Figure S3 shows micro-Raman mapping for the 2D graphene peak, further indicating that graphene film is distributed uniformly on the substrate. Figure 2c shows the high-resolution XPS scan of N 1s centered at 400.7 eV, further confirming that N-doped graphene was obtained under this optimal condition. All the results demonstrate that planar configuration of PAHs precursor containing dopant elements promotes the formation of doped graphene. The atomic concentration of N in TPB-derived doped graphene is about 1.74 % from the data of XPS survey scan. No B 1s peak was observed for this sample, which is probably owing to the difficulty of B–C bonding formation in graphene film at the present condition.

**Fig. 3:**
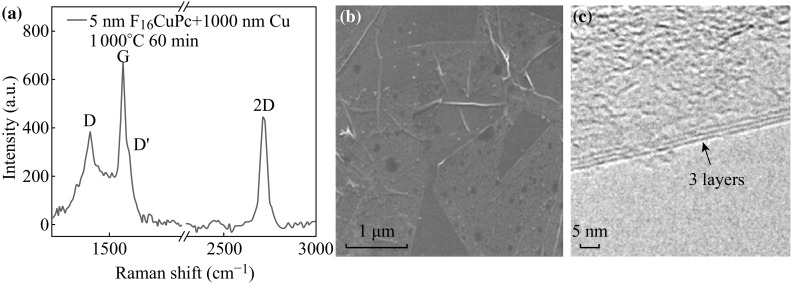
**a** Raman spectrum, **b** SEM image, and **c** HR-TEM image of F_16_CuPc-derived N, F-co-doped graphene

In order to achieve co-doping in graphene by this method, F_16_CuPc of 5 nm was used as the carbon source to prepare N, F-co-doped graphene on the SiO_2_/Si substrate at 1000 °C for 60 min. F_16_CuPc is also a PAH compound with a planar structure. The Raman spectrum of the product (Fig. 3a) shows a large D peak and a small D’ peak, which may be induced by N and F doping atoms. The G/2D ratio is ~1.5 and the 2D peak is lower and broader than that of the single layer graphene, indicating that the obtained doped graphene film is of 3–4 layers. Figure 3b shows SEM image of F_16_CuPc-derived doped graphene. It can be found that thin graphene film is homogeneously distributed on the substrate. Moreover, it can be clearly distinguished from the HR-TEM image shown in Fig. 3c that the doped graphene film is of three layers, which is consistent with Raman analysis.

**Fig. 4:**
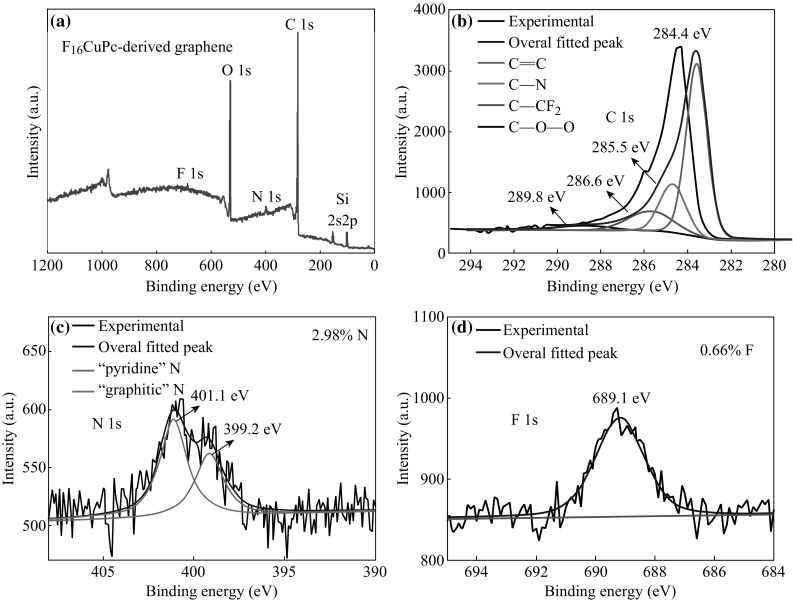
XPS spectroscopic analysis of F_16_CuPc-derived N, F-co-doped graphene. **a** Full XPS spectrum, **b** C 1s, **c** N 1s, and **d** F 1s

XPS investigation further verifies that N and F co-doping has been achieved in the graphene. XPS spectra of N, F-co-doped graphene are shown in Fig. 4. Figure 4a shows the full XPS spectrum of F_16_CuPc-derived doped graphene on the SiO_2_/Si substrate. There is no signal of Cu, indicating the clear removal of Cu after etching. Both the nitrogen- and fluorine-related peaks are obviously found in the survey scan, which confirms the successful co-doping of N and F in the graphene film. The atomic concentration of N and F for F_16_CuPc-derived doped graphene is about 2.98 and 0.66 %, respectively. The characteristic XPS C 1s core-level spectrum (Fig. 4b) is assigned as *sp* ^2^ carbon (284.4 eV), confirming the graphitic structure of the as-grown graphene grains. The shoulder around 285.5 and 286.6 eV can be assigned to the C–N and C–F bonding, respectively. Figure 4c shows the high-resolution XPS scan of N 1s, suggesting two types of N–C bonding: “graphitic” N centered at 401.1 eV and “pyridine” N centered at 399.2 eV [7, 9, 18]. The ratio of two types N indicates that they are mainly bonded to three adjacent carbons, suggesting that the N atoms are uniformly bound to the graphene structure. The high-resolution XPS scan of F 1s shows a single symmetric peak centered at 689.1 eV in Fig. 4d, which is assigned to C–F covalent bond [24].

**Fig. 5:**
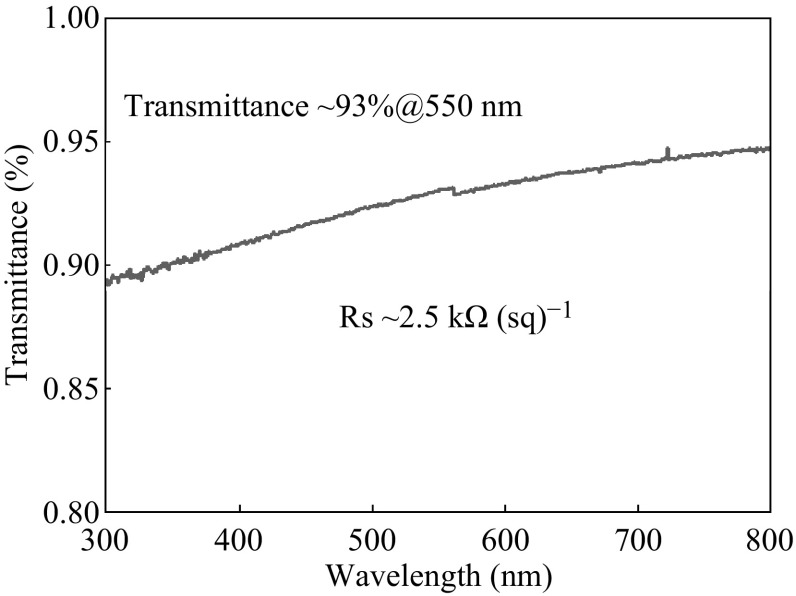
The optical transmittance spectrum of the F_16_CuPc-derived doped graphene on a quartz wafer. *Inset* is the sheet resistance measured by four-point probe measurement

Figure 5 shows the result of optical transmittance measurement for the N, F-co-doped graphene directly grown on quartz in the same condition as using SiO_2_/Si substrate, exhibiting a high optical transmittance of ~93 % at 550 nm, even though the doped graphene film is 3–4 layers. The sheet resistance (*R*_s_) obtained from four-point probe measurement is ∼2.5 kΩ (sq)^−1^, revealing that the as-grown N, F-co-doped graphene film is of high conductivity.

## Conclusions

In summary, a facile method for high-quality synthesis of doped graphene film on the dielectric substrate has been developed. PAHs containing dopant elements with planar configuration were used as both carbon feedstocks and doping sources and a layer of Cu film as the catalyst. The thickness of Cu layer and PAHs, the annealing time, and temperature are optimized for high-quality graphene growth. N-doped and N, F-co-doped graphene have been synthesized using TPB and F_16_CuPc as solid carbon sources, respectively. The properties of the as-grown samples were well studied and N, F-co-doped graphene exhibits a high optical transmittance and low sheet resistance. The present growth strategy provides a controllable transfer-free route for high-quality doped graphene growth, which will facilitate the practical electronic applications of graphene.

## Electronic supplementary material

Below is the link to the electronic supplementary material.

Supplementary material 1 (PDF 473 kb)

## References

[1] Lee CG, Wei XD, Kysar JW, Hone J (2008). Measurement of the elastic properties and intrinsic strength of monolayer graphene. Science.

[2] Du X, Skachko I, Barker A, Andrei EY (2008). Approaching ballistic transport in suspended graphene. Nat. Nanotechnol..

[3] Kim KS, Zhao Y, Jang H, Lee SY, Kim JM, Ahn JH, Kim P, Choi JY, Hong BH (2009). Large-scale pattern growth of graphene films for stretchable transparent electrodes. Nature.

[4] Seol JH, Jo I, Moore AL, Lindsay L, Aitken ZH (2010). Two-dimensional phonon transport in supported graphene. Science.

[5] Sun X, Qiao L, Wang X (2013). A novel immunosensor based on au nanoparticles and polyaniline/multiwall carbon nanotubes/chitosan nanocomposite film functionalized interface. Nano-Micro Lett..

[6] Nair RR, Blake P, Grigorenko AN, Novoselov KS, Booth TJ, Stauber T, Peres NMR, Geim AK (2008). Fine structure constant defines visual transparency of graphene. Science.

[7] Geim AK (2009). Graphene: status and prospects. Science.

[8] Lili Yu, Hui Wu, Beina Wu, Wang Ziyi, Cao Hongmei, Congying Fu, Jia Nengqin (2014). Magnetic Fe_3_O_4_-reduced graphene oxide nanocomposites-based electrochemical biosensing. Nano-Micro Lett..

[9] Xue Y, Wu B, Jiang L, Guo Y, Huang L (2012). Low temperature growth of highly nitrogen-doped single crystal graphene arrays by chemical vapor deposition. J. Am. Chem. Soc..

[10] Haixia W, Liu Q, Guo S (2014). Composites of graphene and LiFePO_4_ as cathode materials for lithium ion battery: a mini-review. Nano-Micro Lett..

[11] Qu LT, Liu Y, Baek JB, Dai LM (2010). Nitrogen-doped graphene as efficient metal-free electrocatalyst for oxygen reduction in fuel cells. ACS Nano.

[12] Yang Z, Yao Z, Li GF, Fang GY, Nie HG, Liu Z, Zhou XM, Chen XA, Huang SM (2011). Sulfur-doped graphene as an efficient metal-free cathode catalyst for oxygen reduction. ACS Nano.

[13] Reddy ALM, Srivastava A, Gowda SR, Gullapalli H, Dubey M, Ajayan PM (2010). Synthesis of nitrogen-doped graphene films for lithium battery application. ACS Nano.

[14] Reina A, Jia XT, Ho J, Nezich D, Son H, Bulovic V, Dresselhaus MS, Kong J (2009). Large area, few-layer graphene films on arbitrary substrates by chemical vapor deposition. Nano Lett..

[15] Lia XS, Caia WW, Ana J, Kimb S, Nahb J (2009). Large-area synthesis of high-quality and uniform graphene films on copper foils. Science.

[16] Luo ZQ, Lim SH, Tian ZQ, Shang JZ, Lai LF, MacDonald B, Fu C, Shen ZX, Yu T, Lin JY (2011). Pyridinic N-doped graphene: synthesis, electronic structure, and electrocatalytic property. J. Mater. Chem..

[17] Jin Z, Yao J, Kittrell C, Tour JM (2011). Large-scale growth and characterizations of nitrogen-doped monolayer graphene sheets. ACS Nano.

[18] Sun Z, Yan Z, Yao J, Beitler E, Zhu Y, Tour JM (2010). Growth of graphene from solid carbon sources. Nature.

[19] Britnell L, Gorbachev RV, Jalil R, Belle BD, Schedin F (2012). Field-effect tunneling transistor based on vertical graphene heterostructures. Science.

[20] Bae S, Kim H, Lee Y, Xu XF, Park JS (2010). Roll-to-roll production of 30-inch graphene films for transparent electrodes. Nat. Nanotechnol..

[21] Zhuo Q, Wang Q, Zhang YP, Zhang D, Li QL (2015). Transfer-free synthesis of doped and patterned graphene films. ACS Nano.

[22] Graf D, Molitor F, Ensslin K, Stampfer C, Jungen A, Hierold C, Wirtz L (2007). Spatially resolved raman spectroscopy of single- and few-layer graphene. Nano Lett..

[23] Ferrari AC, Meyer JC, Scardaci V, Casiraghi C, Lazzeri M (2006). Raman spectrum of graphene and graphene layers. Phys. Rev. Lett..

[24] Stine R, Lee WK, Whitener KE, Robinson JT, Sheehan PE (2013). Chemical stability of graphene fluoride produced by exposure to XeF_2_. Nano Lett..

