# Supplementary material for: Direct Synthesis of Co-doped Graphene on Dielectric Substrates Using Solid Carbon Sources
Source: Nanomicro Lett. 2015 Jul 16;7(4):368–73. doi: 10.1007/s40820-015-0052-6 (PMC6223911; doi:10.1007/s40820-015-0052-6)
Supplement: Supplementary file 1 — Supplementary material 1 (PDF 473 kb) [file 40820_2015_52_MOESM1_ESM.pdf]

Supporting Information for

Direct Synthesis of Co-doped Graphene on Dielectric Substrates Using Solid Carbon Sources

Qi Wang, Pingping Zhang, Qiqi Zhuo, Xiaoxin Lv, Jiwei Wang, Xuhui Sun*

Jiangsu Key Laboratory for Carbon-Based Functional Materials and Devices, Institute of Functional Nano & Soft Materials, Soochow University, Suzhou, Jiangsu, 215123, People's Republic of China

*Corresponding author. E-mail: xhsun@suda.edu.cn

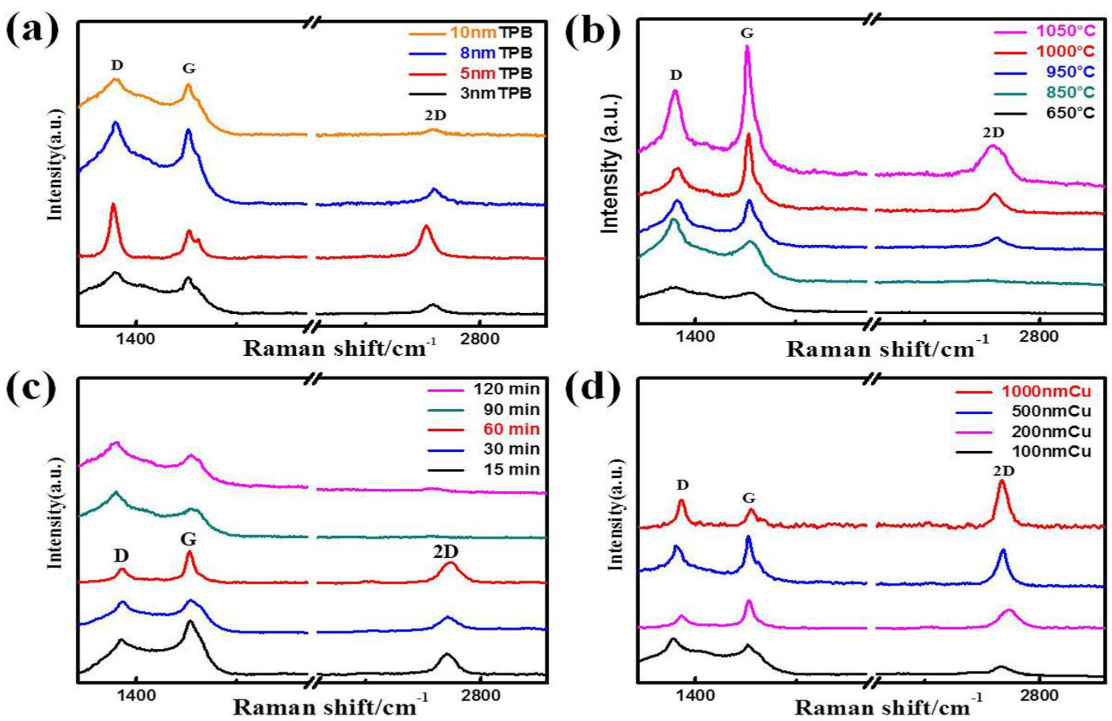

Fig. S1 Raman spectra of TPB-derived doped graphene under different reaction conditions. **a**) Different thickness of solid carbon source: 3, 5, 8, and 10 nm; **b**) Different annealing temperature: 650, 850, 950, 1000, and 1050 °C; **c**) Different annealing time: 15, 30, 60, 90, and 120 min; **d**) Different thickness of Cu film layer: 100, 200, 500, and 1000 nm.

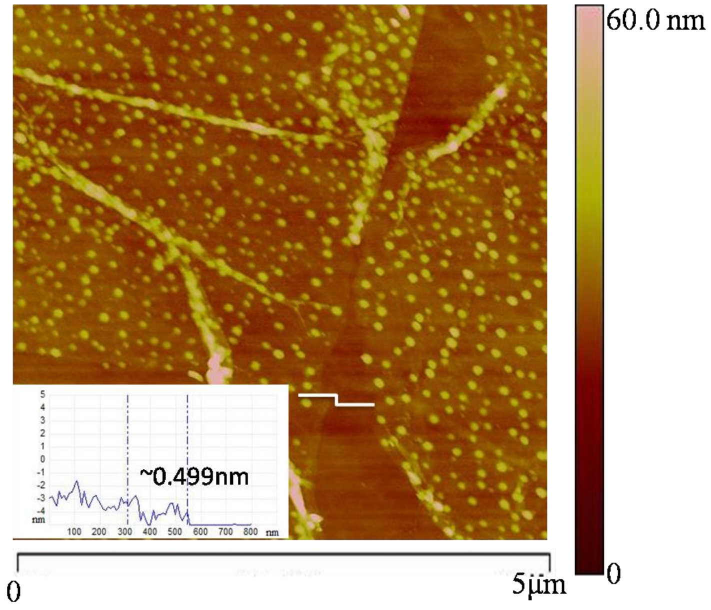

Fig. S2 AFM image of sample with 5 nm TPB, 1000 nm Cu, annealing at 1000 °C for 60 min. The inset image is height distribution, the thickness of as-grown doped-graphene is ~0.499 nm, indicating that the graphene film is monolayer.

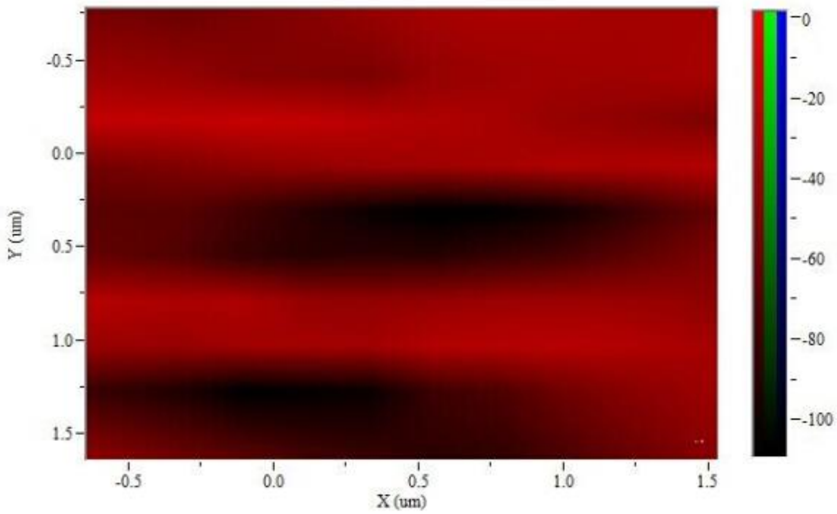

Fig. S3 Micro-Raman mapping for the 2D graphene peak, indicating that graphene film distributes uniformly on the substrate.
